# Ferroptosis drives photoreceptor degeneration in mice with defects in all-*trans*-retinal clearance

**DOI:** 10.1074/jbc.RA120.015779

**Published:** 2020-12-20

**Authors:** Chao Chen, Jingmeng Chen, Yan Wang, Zuguo Liu, Yalin Wu

**Affiliations:** 1 Fujian Provincial Key Laboratory of Ophthalmology and Visual Science, Department of Ophthalmology, Xiang'an Hospital of Xiamen University, Eye Institute of Xiamen University, School of Medicine, Xiamen University, Xiamen City, Fujian, China; 2 School of Medicine, Xiamen University, Xiamen City, Fujian, China; 3 Department of Ophthalmology, Shenzhen Hospital, Southern Medical University, Shenzhen City, Guangdong, China; 4 Xiamen Eye Center of Xiamen University, Xiamen City, Fujian, China; 5 Shenzhen Research Institute of Xiamen University, Shenzhen City, Guangdong, China

**Keywords:** ferroptosis, all-*trans*-retinal, photoreceptor, cell death, lipid peroxidation, oxidative stress, iron metabolism, macular degeneration, Stargardt disease, AMD, age-related macular degeneration, atRAL, all-*trans*-retinal, CP, ceruloplasmin, DMT1, Divalent metal transporter 1, FPN, Ferroportin, GSH, glutathione, H&E, hematoxylin and eosin, IREB2, Iron-responsive element binding protein 2, LDH, lactate dehydrogenase, LED, light emitting diode, ONL, outer nuclear layer, POS, photoreceptor outer segments, ROS, reactive oxygen species, STEAP3, six-transmembrane epithelial antigen of prostate 3, STGD1, autosomal recessive Stargardt disease, TEM, transmission electron microscope, TF, transferrin, TFRC, transferrin receptor

## Abstract

The death of photoreceptor cells in dry age-related macular degeneration (AMD) and autosomal recessive Stargardt disease (STGD1) is closely associated with disruption in all-*trans*-retinal (atRAL) clearance in neural retina. In this study, we reveal that the overload of atRAL leads to photoreceptor degeneration through activating ferroptosis, a nonapoptotic form of cell death. Ferroptosis of photoreceptor cells induced by atRAL resulted from increased ferrous ion (Fe^2+^), elevated ACSL4 expression, system Xc^−^ inhibition, and mitochondrial destruction. Fe^2+^ overload, tripeptide glutathione (GSH) depletion, and damaged mitochondria in photoreceptor cells exposed to atRAL provoked reactive oxygen species (ROS) production, which, together with ACSL4 activation, promoted lipid peroxidation and thereby evoked ferroptotic cell death. Moreover, exposure of photoreceptor cells to atRAL activated COX2, a well-accepted biomarker for ferroptosis onset. In addition to GSH supplement, inhibiting either Fe^2+^ by deferoxamine mesylate salt (DFO) or lipid peroxidation with ferrostatin-1 (Fer-1) protected photoreceptor cells from ferroptosis caused by atRAL. *Abca4*^*−/−*^*Rdh8*^*−/−*^ mice exhibiting defects in atRAL clearance is an animal model for dry AMD and STGD1. We observed that ferroptosis was indeed present in neural retina of *Abca4*^*−/−*^*Rdh8*^*−/−*^ mice after light exposure. More importantly, photoreceptor atrophy and ferroptosis in light-exposed *Abca4*^*−/−*^*Rdh8*^*−/−*^ mice were effectively alleviated by intraperitoneally injected Fer-1, a selective inhibitor of ferroptosis. Our study suggests that ferroptosis is one of the important pathways of photoreceptor cell death in retinopathies arising from excess atRAL accumulation and should be pursued as a novel target for protection against dry AMD and STGD1.

Rhodopsin, a primary photoreceptor molecule in vision, consists of apoprotein opsin and 11-*cis*-retinal (11-*cis*-RAL) chromophore (1, 2). Capture of a photon bleaches rhodopsin and isomerizes 11-*cis*-RAL into all-*trans*-retinal (atRAL), followed by detachment of atRAL from opsin (3, 4). Regeneration of 11-*cis*-RAL from atRAL between photoreceptor and retinal pigment epithelium (RPE) in the visual (retinoid) cycle plays a pivotal role in sustaining vision (5, 6, 7, 8). Although atRAL is an indispensable intermediate of the visual (retinoid) cycle, inadequate or weakened clearance of atRAL resulted in increased levels of atRAL in a free form in the cytosol of photoreceptor outer segments (POS), which is considered to be associated with photoreceptor degeneration in patients with dry age-related macular degeneration (AMD) and autosomal recessive Stargardt disease (STGD1) (9, 10). To avoid the toxicity of atRAL to photoreceptor cells, there must have been proteins or enzymes responsible for incessant clearance of atRAL from POS in the visual (retinoid) cycle (3, 4, 11). ATP-binding cassette transporter 4 (ABCA4) is a retina-specific ABC transporter that involves in shuttling atRAL from POS discs to the cytosol (12, 13, 14) where all-*trans*-retinol dehydrogenase 8 (RDH8) reduces atRAL into all-*trans*-retinol (15). Evidently, the role of ABCA4 and RDH8 in the visual (retinoid) cycle is very critical for clearing atRAL in POS. Mice with a deficiency of *Abca4* and *Rdh8* genes (*Abca4*^*−/−*^*Rdh8*^*−/−*^ mice) display defects in atRAL clearance from POS and mimic basic features of human dry AMD and STGD1, such as photoreceptor and RPE degeneration (9, 16). These lines of evidence imply a direct relationship between atRAL toxicity and photoreceptor atrophy.

Masutomi and coworkers (17) disclose that atRAL evokes photoinduced oxidation in rod photoreceptors. Previous studies also show that atRAL causes cell death in a murine photoreceptor cell line (661W) (18). Most recently, we introduce that activation of c-Jun N-terminal kinase (JNK) promotes photoreceptor apoptosis induced by atRAL, and blocking JNK significantly mitigates photoreceptor atrophy and apoptosis in *Abca4*^*−/−*^*Rdh8*^*−/−*^ mice subjected to light exposure (19). These findings suggest that patients with dry AMD and STGD1 may benefit from the suppression of photoreceptor apoptosis. However, we cannot exclude the possibility that nonapoptotic processes involve the death of photoreceptor cells in dry AMD and STGD1.

In 2012, Stockwell and coworkers reported ferroptosis, a unique form of nonapoptotic cell death (20). Ferroptosis, different from apoptosis, necrosis, and autophagic cell death based on morphological, biochemical, and genetical criteria (20), features lipid peroxidation and depends on iron and lipid-based reactive oxygen species (lipid ROS) (20, 21, 22). Several lines of investigation have identified *prostaglandin-endoperoxide synthase 2* (*Ptgs2*) and its gene product cyclooxygenase 2 (COX2) as downstream molecular markers of ferroptosis (23, 24). Acyl-CoA synthetase long-chain family member 4 (ACSL4) is a convertor of free fatty acids into fatty CoA esters (25). The induction of ACSL4 contributes significantly to ferroptosis execution by catalyzing the formation of polyunsaturated fatty acid (PUFA)-containing phospholipids required for the production of lipid peroxidation products (26, 27). System Xc^−^ is a cystine–glutamate antiporter that affords sufficient levels of cystine used for the synthesis of antioxidant tripeptide glutathione (GSH) in nearly all living cells (28). Inhibition of system Xc^−^ activity by erastin in cancer cells triggers ferroptosis and results in a compensatory transcriptional upregulation of solute carrier family 7 membrane 11 (SLC7A11), a key component of system Xc^−^ (20, 29). In addition, ferroptotic cancer cells exhibit smaller than normal mitochondria with increased membrane density and collapsed mitochondrial cristae (20). Besides cancer cell death, ferroptosis is also implicated in pathological processes of kidney failure, stroke, intestinal ischemia/reperfusion injury, Parkinson's disease, and Alzheimer's disease (30, 31, 32, 33, 34, 35, 36). Nevertheless, it remains unknown whether ferroptosis mediates dry AMD and STGD1 and whether atRAL functions as a physiological trigger for inducing ferroptosis of photoreceptor cells. In the present study, we elucidated the role of ferroptosis in photoreceptor degeneration of retinopathies resulting from disrupted clearance of atRAL.

## Results

### atRAL stimulates ferroptosis in 661W photoreceptor cells

**Figure 1:**
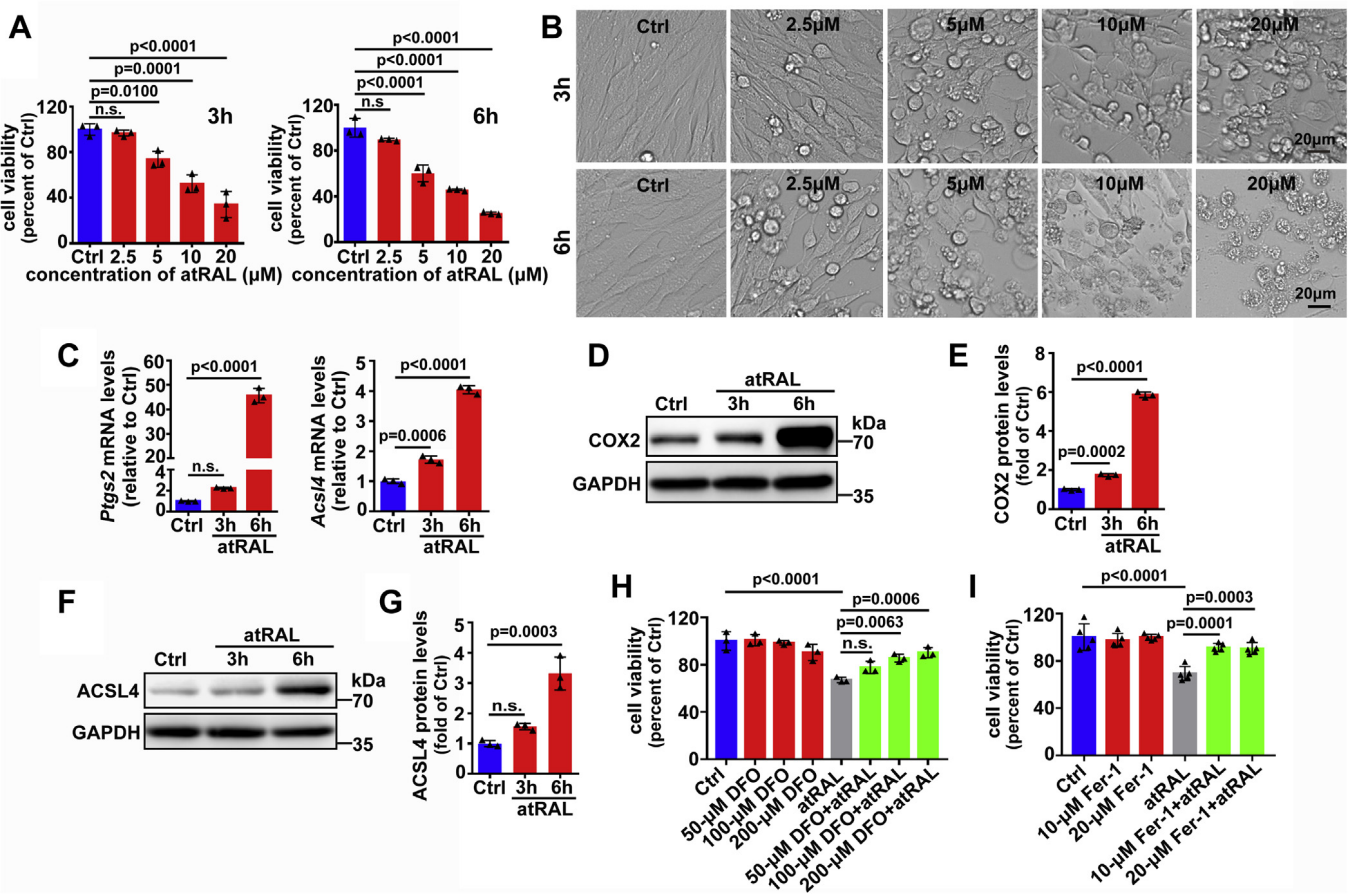
**atRAL induces ferroptosis in 661W photoreceptor cells.***A*, cell viability, 3 and 6 h after incubating 661W photoreceptor cells with serial concentrations of atRAL (2.5, 5, 10, and 20 μM), was probed by MTS assay. *B*, cellular morphology, 3 and 6 h after incubating 661W photoreceptor cells with atRAL at concentrations of 2.5, 5, 10, and 20 μM, was imaged by a Leica DMi8 inverted microscope. *Scale bars*, 20 μm. *C*, qRT-PCR analysis of *Ptgs2* and *Acsl4* mRNA levels in 661W photoreceptor cells treated with 5-μM atRAL for 3 and 6 h. *D*–*G*, western blot analysis of COX2 and ACSL4 in 661W photoreceptor cells exposed to 5-μM atRAL for 3 and 6 h. Protein levels of COX2 and ACSL4 were normalized to those of GAPDH and presented as fold changes relative to vehicle (DMSO) controls. *H*, cytotoxicity was examined by MTS assay. 661W photoreceptor cells were pretreated with iron chelating agent DFO (50, 100, and 200 μM) for 2 h, followed by incubation with or without 5-μM atRAL for 6 h, respectively. Cells treated with atRAL or vehicle (DMSO) alone served as controls. *I*, cell viability was assessed by MTS assay. 661W photoreceptor cells were preincubated with Fer-1 (10 and 20 μM) for 2 h, followed by treatment with or without 5-μM atRAL for 6 h, respectively. Control cells were treated with atRAL or vehicle (DMSO) alone. Molecular mass markers (kDa) were indicated to the *right* of immunoblots. n.s., not significant.

661W is a cell line for cone photoreceptors that is derived from a mouse retinal tumor and serves as an *in vitro* model for studying retinal degeneration (37). The results of the MTS assay shown in Figure 1*A* demonstrated that atRAL diminished the viability of 661W photoreceptor cells in a concentration- and time-dependent manner. Treating 661 W photoreceptor cells with atRAL for 3 and 6 h at a concentration of 5 μM gave rise to significant decreases in cell viability of approximately 26.3 and 39.8%, respectively. When exposed to atRAL for 3 and 6 h at concentrations starting from 2.5 μM, 661W photoreceptor cells exhibited altered morphology, which is characterized by rounding, shrinkage, and cytoplasmic rupture (Fig. 1*B*). On the basis of MTS data and morphological images, atRAL at the concentration of 5 μM was used to treat 661W photoreceptor cells for 3 or 6 h in subsequent experiments. Evidence from our recent research has indicated that 5-μM atRAL triggers apoptosis of 661W photoreceptor cells after 6 h of treatment (19). However, to further ascertain whether atRAL-loaded photoreceptor cells suffer from nonapoptotic forms of cell death, we used quantitative reverse transcription–polymerase chain (qRT-PCR) and western blotting to analyze the expression of recognized ferroptosis-related genes and proteins, respectively. Interestingly, 5-μM atRAL significantly and time-dependently increased mRNA levels of *Ptgs2* and *Acsl4* in 661W photoreceptor cells (Fig. 1*C*). Immunoblot analysis also indicated that 5-μM atRAL elicited a significant time-dependent elevation in protein levels of COX2 encoded by *Ptgs2* gene in lysates of 661W photoreceptor cells (Fig. 1, *D*–*E*). By contrast, protein expression of ACSL4 in lysates of atRAL-treated 661W photoreceptor cells showed a trend of increase from 3 to 6 h, but manifested statistical significance at 6 h (Fig. 1, *F*–*G*). Deferoxamine mesylate salt (DFO) is a ferrous ion (Fe^2+^) chelator that relieves ferroptotic cell death (20). The results of MTS assay showed that treatment with DFO at concentrations of 50, 100, and 200 μM concentration-dependently and effectively protected 661W photoreceptor cells from ferroptosis caused by 5-μM atRAL (Fig. 1*H*). More importantly, treatment with 10- and 20-μM ferrostatin-1 (Fer-1), a selective ferroptosis inhibitor (38, 39), notably improved the viability of 661W photoreceptor cells incubated for 6 h with 5-μM atRAL (Fig. 1*I*). These findings reveal the ability of atRAL to incite ferroptosis of photoreceptor cells.

### atRAL causes iron dyshomeostasis in 661W photoreceptor cells

**Figure 2:**
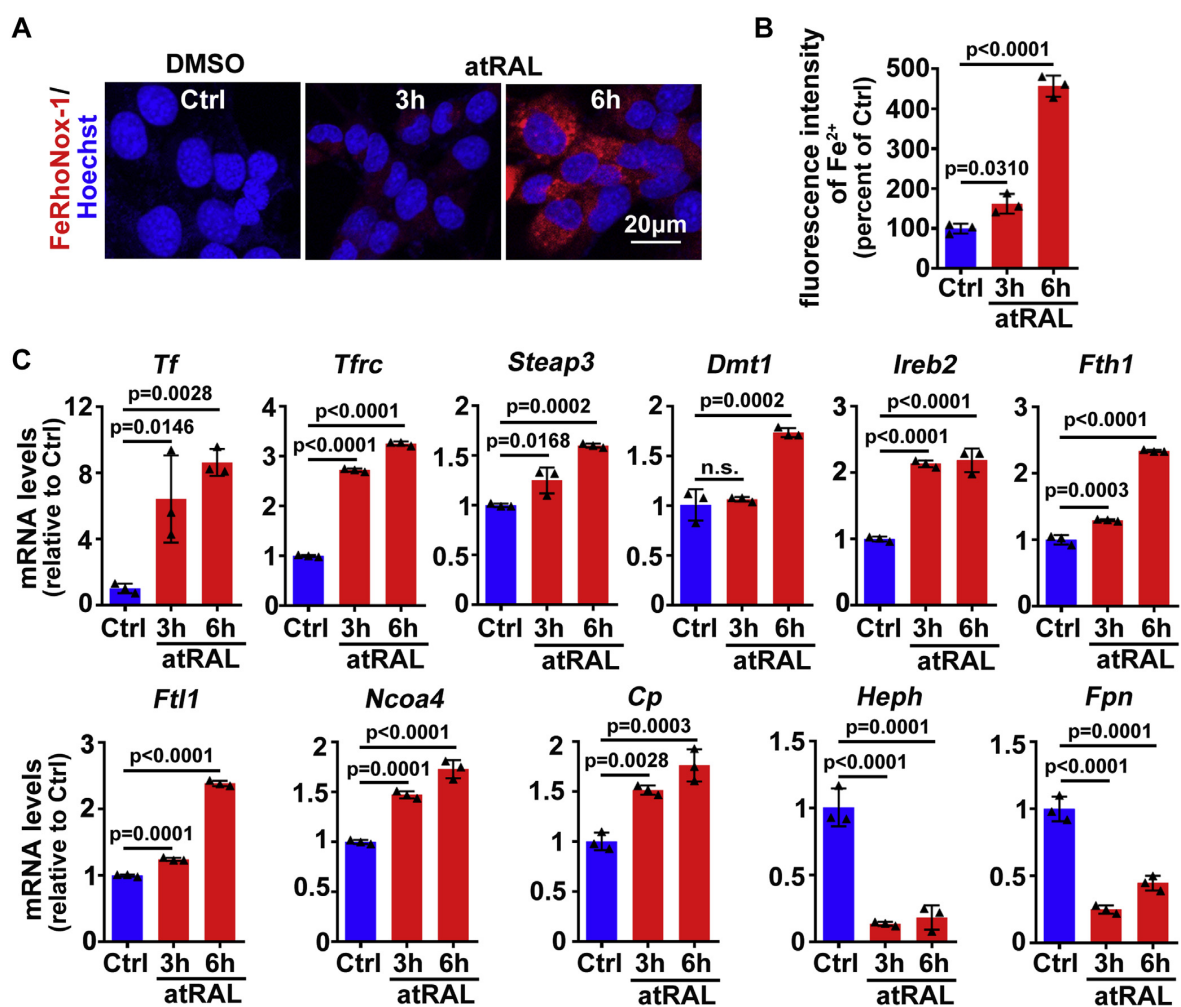
**atRAL disrupts iron homeostasis in 661W photoreceptor cells.***A*, intracellular Fe^2+^, 3 and 6 h after incubating 661W photoreceptor cells with 5-μM atRAL, was visualized by FeRhoNox-1 staining coupled with confocal microscopy. Nuclei were stained *blue* with Hoechst 33342. *Scale bars*, 20 μm. *B*, quantification of fluorescence intensity of Fe^2+^ specifically detected by FeRhoNox-1 in 661W photoreceptor cells treated with 5-μM atRAL for 3 and 6 h. *C*, qRT-PCR analysis of iron homeostasis-related genes in 661W photoreceptor cells exposed to 5-μM atRAL for 3 and 6 h. Control cells were treated with vehicle (DMSO) alone for 6 h. n.s., not significant.

Excess Fe^2+^, which exhibits high cytotoxicity *via* the Fenton reaction, can facilitate ferroptotic cell death (20, 40). Imaging of Fe^2+^ using FeRhoNox-1 showed that 5-μM atRAL dramatically enhanced intracellular Fe^2+^ levels in 661W photoreceptor cells at 3 to 6 h of exposure (Fig. 2, *A*–*B*). The mechanisms by which cellular iron homeostasis is modulated have been well elucidated (41, 42). Transferrin (TF) captures ferric iron (Fe^3+^) and binds to transferrin receptor (TFRC). Cellular uptake of iron, which maintains intracellular iron pool, is accomplished by TFRC–mediated endocytosis of the complex into specialized endosomes where Fe^3+^ is reduced to Fe^2+^ by six-transmembrane epithelial antigen of prostate 3 (STEAP3). Divalent metal transporter 1 (DMT1) serves to deliver Fe^2+^ from the endosomes to cytoplasm where some of Fe^2+^ is stored in ferritin including ferritin heavy chain 1 (FTH1) and ferritin light chain 1 (FTL1). Instead, ferritinophagy modulated by a cargo protein called nuclear receptor coactivator 4 (NOCA4) leads to ferritin degradation and thereby releases Fe^2+^. Ferroportin (FPN) moves Fe^2+^ out of the cells in collaboration with ferroxidases ceruloplasmin (CP) or hephaestin (HEPH). Iron-responsive element binding protein 2 (IREB2) is an RNA binding protein that regulates transcript stability (43, 44). A previous study has revealed that knockdown of IREB2 expression by RNA interference can restrict erastin-induced ferroptosis (20). In the current investigation, mRNA levels of iron homeostasis-related genes in atRAL-loaded 661W photoreceptor cells were examined by qRT-PCR. We observed that treatment with 5-μM atRAL significantly upregulated mRNA levels of *Tf*, *Tfrc*, *Steap3*, *Dmt1*, *Ireb2*, *Fth1*, *Ftl1*, *Ncoa4*, and *Cp*, but clearly downregulated the expression of *Heph* and *Fpn* genes (Fig. 2*C*). These results imply that atRAL disrupts iron homeostasis and thereby increases Fe^2+^, which may contribute to photoreceptor ferroptosis.

### atRAL evokes lipid peroxidation in 661W photoreceptor cells

**Figure 3:**
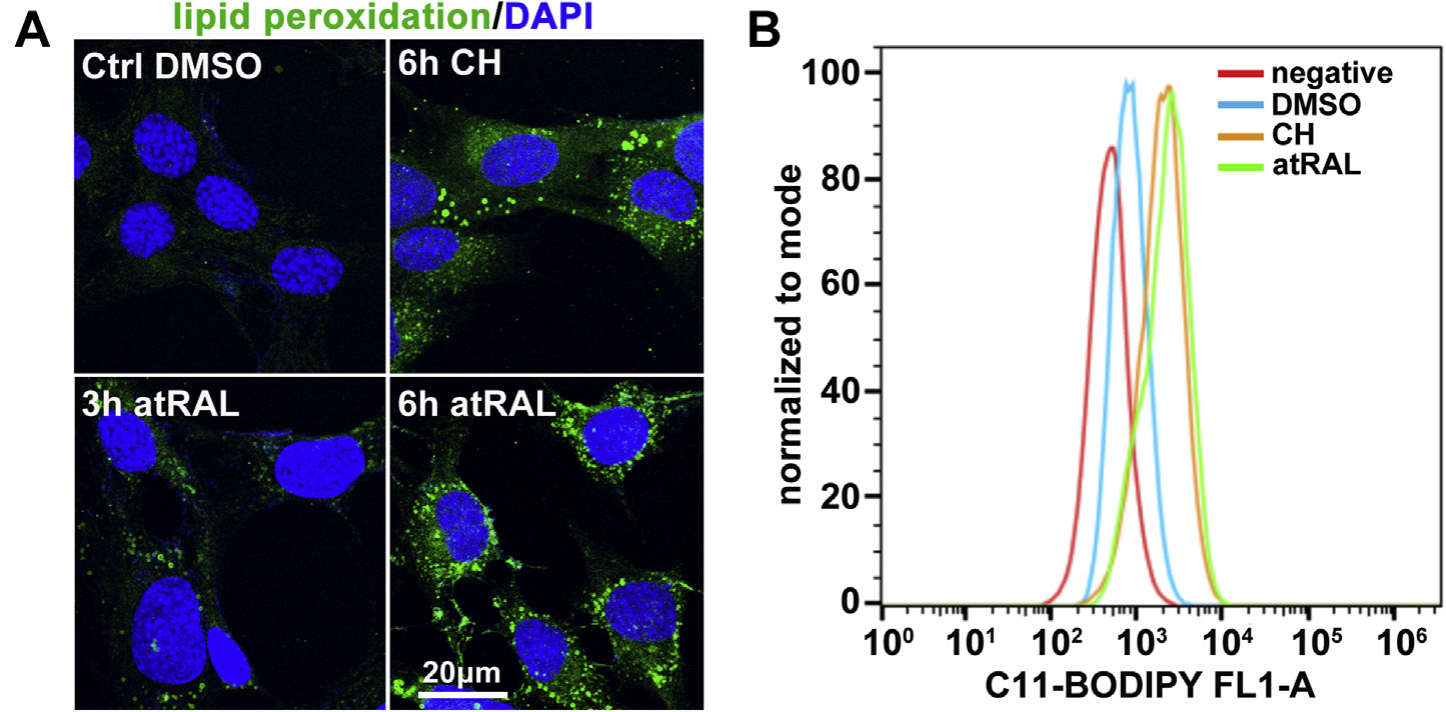
**atRAL causes lipid peroxidation in 661W photoreceptor cells.***A*, lipid peroxidation, 3 and 6 h after exposure of 661W photoreceptor cells to 5-μM atRAL, was examined by an Olympus FV1000 confocal fluorescence microscope using Click-iT lipid peroxidation imaging kit. Nuclei were stained with DAPI (*blue*). *Scale bars*, 20 μm. *B*, flow cytometry coupled with C11-BODIPY staining was used to determine levels of lipid peroxides in 661W photoreceptor cells treated with 5-μM atRAL for 6 h. Cells incubated for 6 h with 100-μM cumene hydroperoxide (CH), a compound that stimulates lipid peroxidation, served as a positive control. Control cells were treated with vehicle (DMSO) alone for 6 h.

One of critical ferroptosis hallmarks is lipid peroxidation (20, 21, 45). The interplay between lipids and intracellular ROS represents a fundamental process in ferroptosis (20, 46, 47). Evidence from our recently published study has revealed that intracellular ROS production is substantially elevated in 661W photoreceptor cells at 6 h after exposure to 5-μM atRAL (19). In this study, we measured the levels of lipid peroxidation in 661W photoreceptor cells treated for 3 and 6 h with 5-μM atRAL by a Click-iT lipid peroxidation imaging kit (ThermoFisher Scientific; Rockford, IL, USA). Imaging by confocal laser scanning microscopy manifested that atRAL significantly and time-dependently aggravated lipid peroxidation within 661W photoreceptor cells (Fig. 3*A*). Moreover, we employed C11-BODIPY staining and flow cytometry to quantify lipid peroxidation in 661W photoreceptor cells exposed to 5-μM atRAL for 6 h and found that levels of lipid peroxides remarkably increased (Fig. 3*B*). Based on these findings, we conclude that atRAL provokes photoreceptor ferroptosis by accentuating lipid peroxidation.

### GSH depletion by atRAL promotes ferroptosis of 661W photoreceptor cells

**Figure 4:**
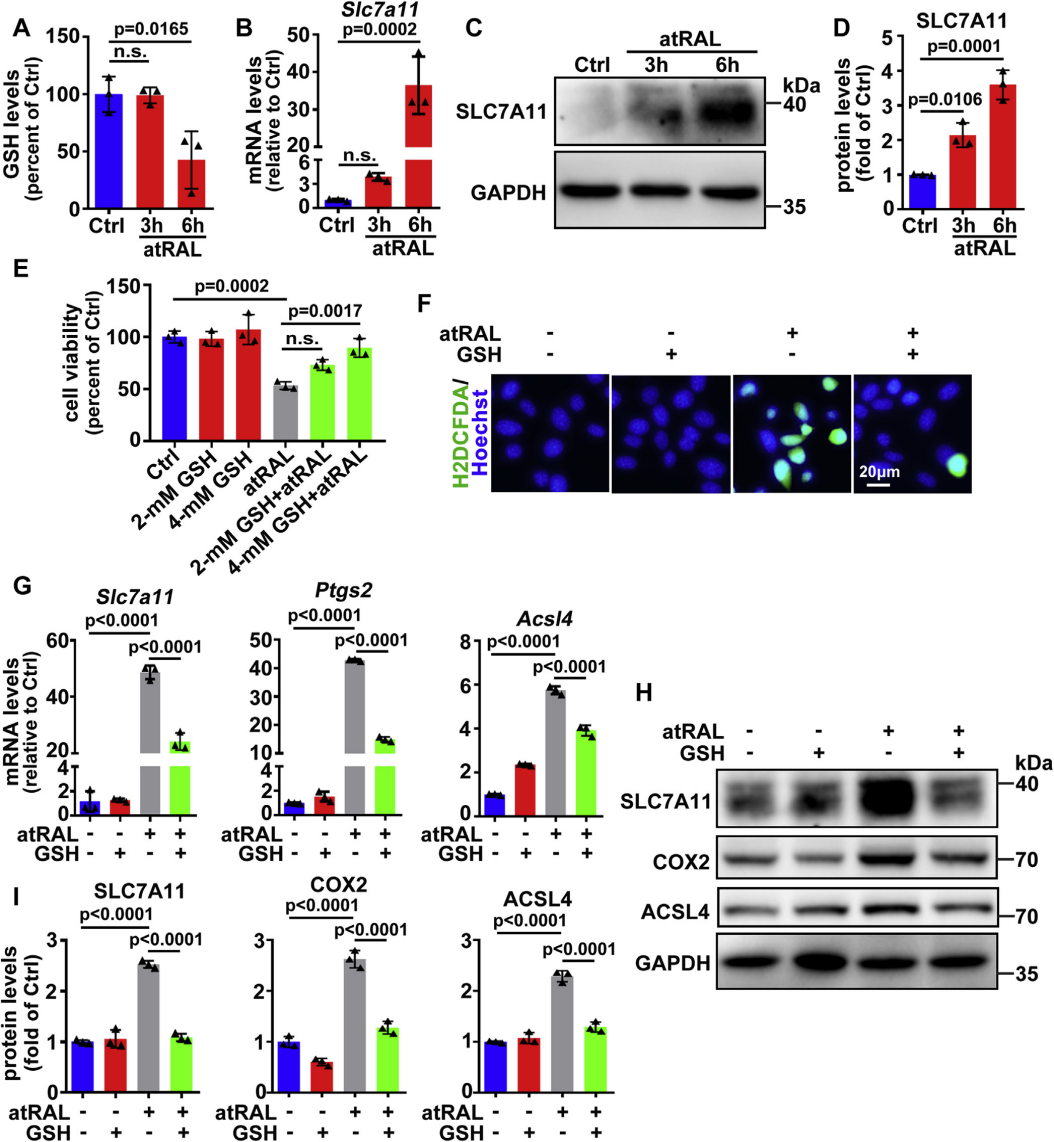
**GSH depletion promotes atRAL-induced ferroptosis in 661W photoreceptor cells.***A*, GSH levels, 3 and 6 h after incubating 661W photoreceptor cells with 5-μM atRAL, were determined using a GSH assay kit and shown as the percentage of GSH content in control cells. *B*, mRNA levels of *Slc7a11*, 3 and 6 h after incubating 661W photoreceptor cells with 5-μM atRAL, were determined using qRT-PCR. *C*, western blot analysis of SLC7A11 in 661W photoreceptor cells exposed to 5-μM atRAL for 3 and 6 h. *D*, protein levels of SLC7A11 were normalized to those of GAPDH, and presented as fold changes relative to vehicle (DMSO) controls. *E*, cell viability was probed by MTS assay. 661W photoreceptor cells were pretreated with 2 or 4-mM GSH for 1 h, followed by incubation with or without 5-μM atRAL for 6 h, respectively. *F*, intracellular ROS generation was visualized using the fluorescent probe H2DCFDA by fluorescence microscopy. 661W photoreceptor cells were pretreated with 4-mM GSH for 1 h and incubated with or without 5-μM atRAL for 6 h. Nuclei were stained with Hoechst 33342 (*blue*). *Scale bars*, 20 μm. *G*, qRT-PCR analysis of mRNA levels of *Slc7a11*, *Ptgs2*, and *Acsl4* in lysates of 661W photoreceptor cells incubated with 5-μM atRAL for 6 h in the presence or absence of 4-mM GSH. Note that cells were pretreated with GSH for 1 h. *H*, western blot analysis of SLC7A11, COX2, and ACSL4 in lysates of 661W photoreceptor cells incubated with 5-μM atRAL for 6 h with or without 4-mM GSH. Note that cells were pretreated with GSH for 1 h. *I*, protein levels of SLC7A11, COX2, and ACSL4 were normalized to those of GAPDH, and presented as fold changes relative to vehicle (DMSO) controls. Cells treated with atRAL, GSH, or vehicle (DMSO) alone served as controls. n.s., not significant.

GSH is indispensable for physiologically defensive responses against oxidative stress (48), and its depletion is also regarded as a cause of ferroptosis onset (20, 49). We found that GSH levels were notably reduced in 661W photoreceptor cells after 6 h of exposure to 5-μM atRAL (Fig. 4*A*). SLC7A11 is a crucial constituent of the cystine/glutamine antiporter (system X_C_^−^), yet an important responsibility of system X_C_^−^ is to generate GSH (50). Former researches have indicated that disturbed system X_C_^−^ leads to GSH depletion and thereby evokes ferroptotic cell death as well as compensatorily elevated gene and protein expression of SLC7A11 (51, 52). As expected, time-dependent increases in mRNA and protein levels of SLC7A11 were observed in 661W photoreceptor cells treated with 5-μM atRAL (Fig. 4, *B*–*D*). In atRAL-loaded 661W photoreceptor cells, increased expression of *Slc7a11* gene was statistically significant at 6 h (Fig. 4*B*), but starting from 3 h, atRAL remarkably enhanced protein expression of SLC7A11 (Fig. 4, *C*–*D*). As revealed by MTS assay, GSH concentration-dependently rescued 661W photoreceptor cells against cell death caused by 5-μM atRAL, and treatment with GSH at a concentration of 4 mM significantly improved cell viability (Fig. 4*E*). On examination by fluorescence microscopy in combination with the fluorescent probe H2DCFDA, 4-mM GSH effectively reduced ROS production in 661W photoreceptor cells incubated with 5-μM atRAL for 6 h (Fig. 4*F*). More importantly, treatment with 4-mM GSH clearly decreased mRNA levels of *Slc7a11*, *Ptgs2*, and *Acsl4* genes as well as protein expression of SLC7A11, COX2, and ACSL4 in 661W photoreceptor cells after 6 h of exposure to 5-μM atRAL (Fig. 4, *G*–*I*). These results testify that GSH depletion induced by atRAL facilitates photoreceptor ferroptosis.

### atRAL disrupts mitochondria in 661W photoreceptor cells

**Figure 5:**
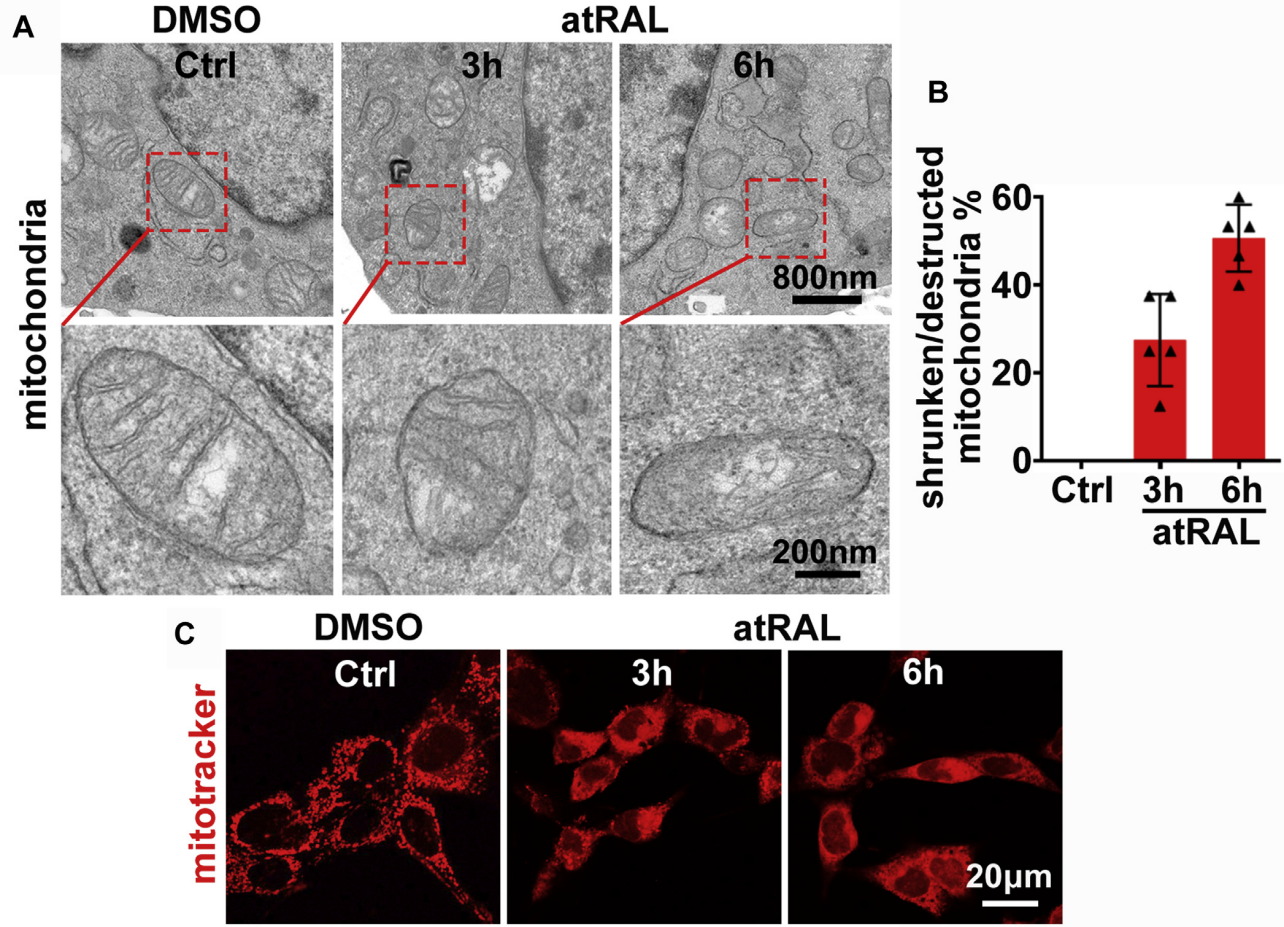
**atRAL causes mitochondrial dyshomeostasis in 661W photoreceptor cells.***A*, TEM images of mitochondria in 661W photoreceptor cells treated with 5-μM atRAL for 3 and 6 h. *Scale bars*, 800 nm (*upper panels*) and 200 nm (*lower panels*). *B*, percentage of shrunken/destructed mitochondria in 661W photoreceptor cells exposed to 5-μM atRAL for 3 and 6 h. *C*, mitochondria in 661W photoreceptor cells exposed to 5-μM atRAL for 3 and 6 h were examined by confocal microscopy after cell staining with MitoTracker Red CMXRos. Cells incubated with vehicle (DMSO) alone for 6 h served as a control. *Scale bars*, 20 μm.

When examined under transmission electron microscope (TEM), the mitochondria in 661W photoreceptor cells treated with 5-μM atRAL for 3 and 6 h exhibited reduction in size and loss of cristae (Fig. 5*A*). These changes in morphology and ultrastructure of mitochondria in atRAL-loaded 661W photoreceptor cells are also observed in cells undergoing ferroptosis (20, 23, 24). The number of shrunken/destructed mitochondria accounted for ∼27.5% of total mitochondria at 3 h after exposure of 661W photoreceptor cells to 5-μM atRAL and increased to ∼50.6% of total mitochondria following 6 h of treatment (Fig. 5*B*). We further analyzed the distribution and morphology of mitochondria in atRAL-exposed 661W photoreceptor cells by Mitotracker Red CMXRos staining and found that mitochondria clearly huddled together in the cytosol compared to normal mitochondria that dispersed homogeneously to form a filamentous reticular network (Fig. 5*C*). Taken together, these findings suggest that atRAL may elicit ferroptosis of photoreceptor cells by damage to mitochondria.

### Inhibiting Fe^2+^ mitigates photoreceptor ferroptosis mediated by atRAL

**Figure 6:**
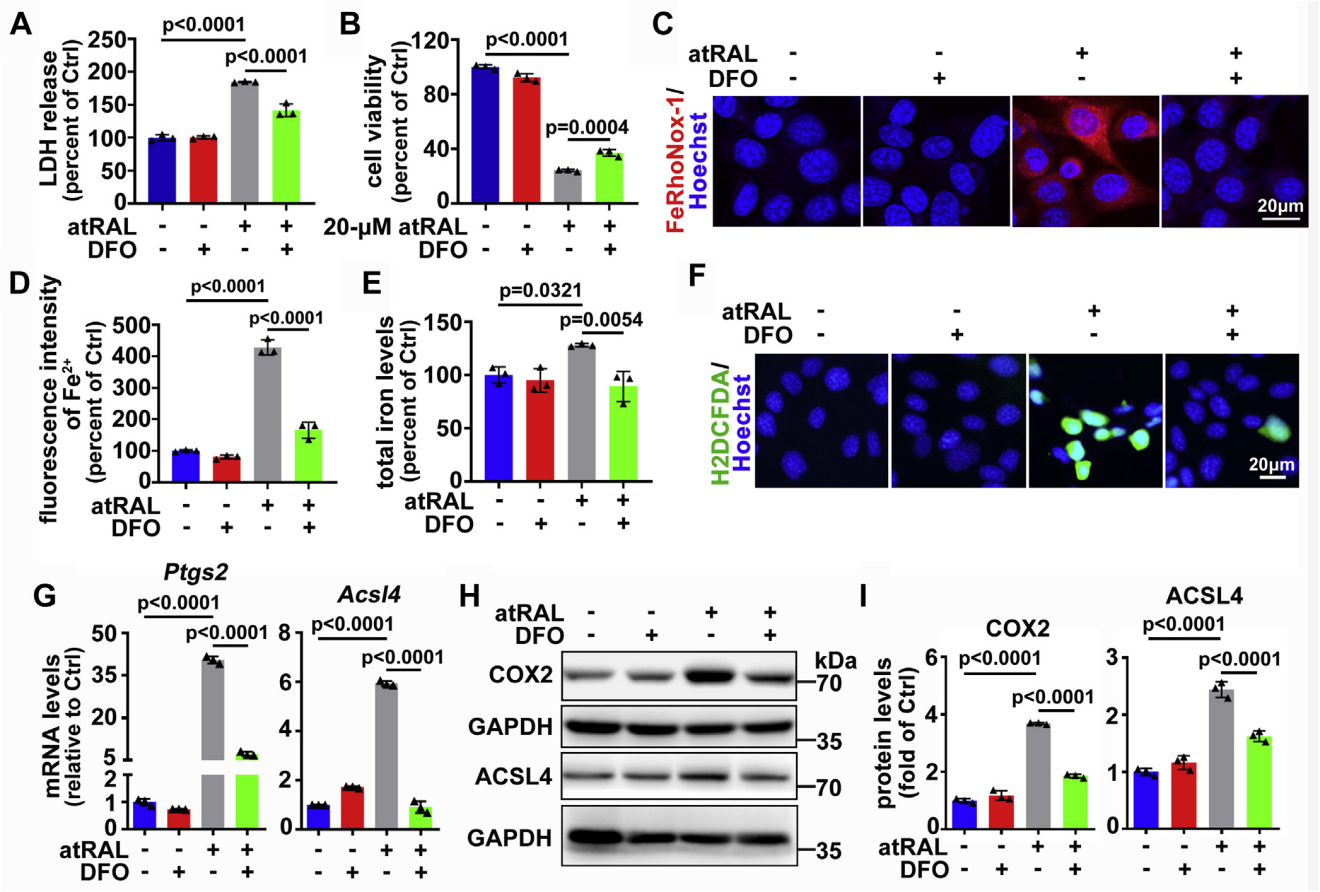
**Iron-chelating agent DFO relieves atRAL-induced ferroptosis in 661W photoreceptor cells.***A*, cytotoxicity was evaluated by LDH release assay. 661W photoreceptor cells were pretreated with 200-μM DFO for 2 h and then incubated for 6 h with 5-μM atRAL. *B*, cell viability was examined by MTS assay. 661W photoreceptor cells were pretreated with 200-μM DFO for 2 h and then incubated with 20-μM atRAL for 6 h. *C*, confocal analysis of intracellular Fe^2+^ with the fluorescent probe FeRhoNox-1. 661W photoreceptor cells were pretreated with 200-μM DFO for 2 h and then incubated for 6 h with 5-μM atRAL. Nuclei were stained with Hoechst 33342 (*blue*). *Scale bars*, 20 μm. *D*, quantification of fluorescence intensity of Fe^2+^ specifically detected by FeRhoNox-1 in 661W photoreceptor cells treated with 5-μM atRAL for 6 h in the presence of 200-μM DFO. Note that cells were pretreated with DFO for 2 h. *E*, total iron levels were analyzed by ICP-MS. 661W photoreceptor cells were pretreated with 200-μM DFO for 2 h and then incubated for 6 h with 5-μM atRAL. *F*, the generation of intracellular ROS was visualized using the fluorescent probe H2DCFDA by fluorescence microscopy. 661W photoreceptor cells were pretreated with 200-μM DFO for 2 h and then incubated with 5-μM atRAL for 6 h. Nuclei were stained *blue* with Hoechst 33342. *Scale bars*, 20 μm. *G*, qRT-PCR analysis of mRNA levels of *Ptgs2* and *Acsl4* in 661W photoreceptor cells treated with 5-μM atRAL for 6 h in the presence of 200-μM DFO. Note that cells were pretreated with DFO for 2 h. *H*, immunoblot analysis of COX2 and ACSL4 in 661W photoreceptor cells exposed to 5-μM atRAL for 6 h with 200-μM DFO. Note that cells were pretreated with DFO for 2 h. *I*, protein levels of COX2 and ACSL4 were normalized to those of GAPDH, and presented as fold changes relative to vehicle (DMSO) controls. Cells treated with atRAL, DFO, or vehicle (DMSO) alone served as controls.

Analysis of membrane integrity by lactate dehydrogenase (LDH) release assay demonstrated that 200-μM DFO significantly prevented LDH release from 661W photoreceptor cells exposed to 5-μM atRAL for 6 h (Fig. 6*A*). Interestingly, the viability of 661W photoreceptor cells after 6 h of exposure to atRAL at a much higher concentration (20 μM) was still increased from 24.2% to 37.1% by 200-μM DFO (Fig. 6*B*). Confocal microscopy with FeRhoNox-1 staining revealed that 200-μM DFO significantly decreased the levels of Fe^2+^ induced by 5-μM atRAL in 661W photoreceptor cells (Fig. 6, *C*–*D*). Quantification of iron by inductively coupled plasma mass spectrometry (ICP-MS) also showed that treatment with 200-μM DFO dramatically reduced total iron levels in atRAL-loaded 661W photoreceptor cells (Fig. 6*E*). Moreover, 200-μM DFO significantly attenuated the production of ROS in 661W photoreceptor cells after 6 h of exposure to 5-μM atRAL (Fig. 6*F*). As expected, qRT-PCR analysis indicated that treatment with 200-μM DFO led to a remarkable decline in mRNA levels of *Ptgs2* and *Acsl4* in 661W photoreceptor cells exposed to 5-μM atRAL for 6 h (Fig. 6*G*). Western blot analysis also demonstrated that 200-μM DFO clearly decreased protein levels of COX2 and ACSL4 in atRAL-loaded 661W photoreceptor cells (Fig. 6, *H*–*I*). These results provide evidence that atRAL-induced death of 661W photoreceptor cells depends on iron, and inhibition of Fe^2+^ significantly rescues photoreceptor cells against ferroptosis resulting from atRAL.

### Suppression of ferroptosis by Fer-1 protects 661W photoreceptor cells against atRAL exposure

**Figure 7:**
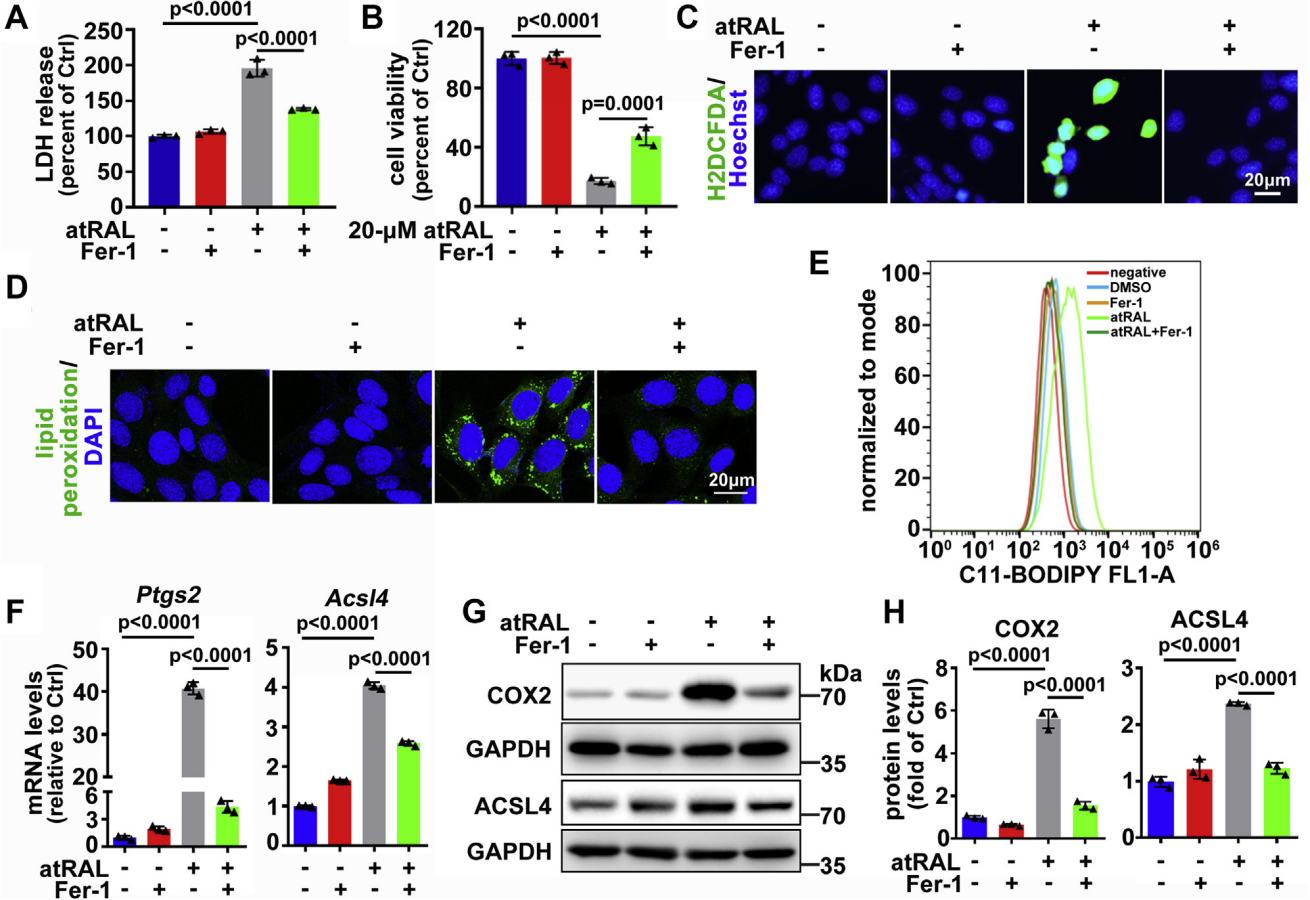
**Ferroptosis inhibitor Fer-1 mitigates atRAL-induced ferroptosis in 661W photoreceptor cells.***A*, cytotoxicity was examined by LDH release assay. 661W photoreceptor cells were pretreated with 20-μM Fer-1 for 2 h and then incubated for 6 h with 5-μM atRAL. *B*, cell viability was determined by MTS assay. 661W photoreceptor cells were pretreated with 20-μM Fer-1 for 2 h and then incubated with 20-μM atRAL for 6 h. *C*, the production of intracellular ROS was visualized by use of the fluorescent probe H2DCFDA and fluorescence microscopy. 661W photoreceptor cells were pretreated with 20-μM Fer-1 for 2 h and then incubated with 5-μM atRAL for 6 h. Nuclei were stained *blue* with Hoechst 33342. *Scale bars*, 20 μm. *D*, lipid peroxidation was measured with Click-iT lipid peroxidation imaging kit and imaged on the confocal fluorescence microscope. 661W photoreceptor cells were pretreated with 20-μM Fer-1 for 2 h and then incubated with 5-μM atRAL for 6 h. Nuclei were stained *blue* by DAPI. *Scale bars*, 20 μm. *E*, levels of lipid peroxides in 661W photoreceptor cells treated with 5-μM atRAL for 6 h with 20-μM Fer-1 were assessed by C11-BODIPY staining coupled with flow cytometry. Note that cells were pretreated with Fer-1 for 2 h. *F*, qRT-PCR analysis of mRNA levels of *Ptgs2* and *Acsl4* in 661W photoreceptor cells exposed to 5-μM atRAL for 6 h in the presence of 20-μM Fer-1. Note that cells were pretreated with Fer-1 for 2 h. *G*, western blot analysis of COX2 and ACSL4 in 661W photoreceptor cells treated with 5-μM atRAL for 6 h with 20-μM Fer-1. Note that cells were pretreated with Fer-1 for 2 h. *H*, protein levels of COX2 and ACSL4 were normalized to those of GAPDH, and presented as fold changes relative to vehicle (DMSO) controls. Cells treated with atRAL, Fer-1, or vehicle (DMSO) alone served as controls.

The results of LDH release assay revealed that Fer-1 at a concentration of 20 μM dramatically decreased the release of LDH from 661W photoreceptor cells after 6 h of exposure to 5-μM atRAL (Fig. 7*A*). By contrast, the viability of 661W photoreceptor cells following 6 h of exposure to 20-μM atRAL was also increased from 17.2% to 47.3% by 20-μM Fer-1 (Fig. 7*B*). Moreover, 20-μM Fer-1 significantly attenuated ROS production and lipid peroxidation caused by 5-μM atRAL in 661W photoreceptor cells (Fig. 7, *C*–*E*). The qRT-PCR analysis manifested that 20-μM Fer-1 significantly reduced mRNA levels of *Ptgs2* and *Acsl4* in 661W photoreceptor cells after 6 h of exposure to 5-μM atRAL (Fig. 7*F*). Likewise, immunoblot analysis indicated that protein levels of COX2 and ACSL4 were dramatically downregulated by 20-μM Fer-1 in atRAL-loaded 661W photoreceptor cells (Fig. 7, *G*–*H*). These findings imply that attenuation of ferroptosis ameliorates the toxicity of atRAL to photoreceptor cells.

### Photoinduced photoreceptor degeneration in *Abca4*^*−/−*^*Rdh8*^*−/−*^ mice implicates ferroptotic cell death

**Figure 8:**
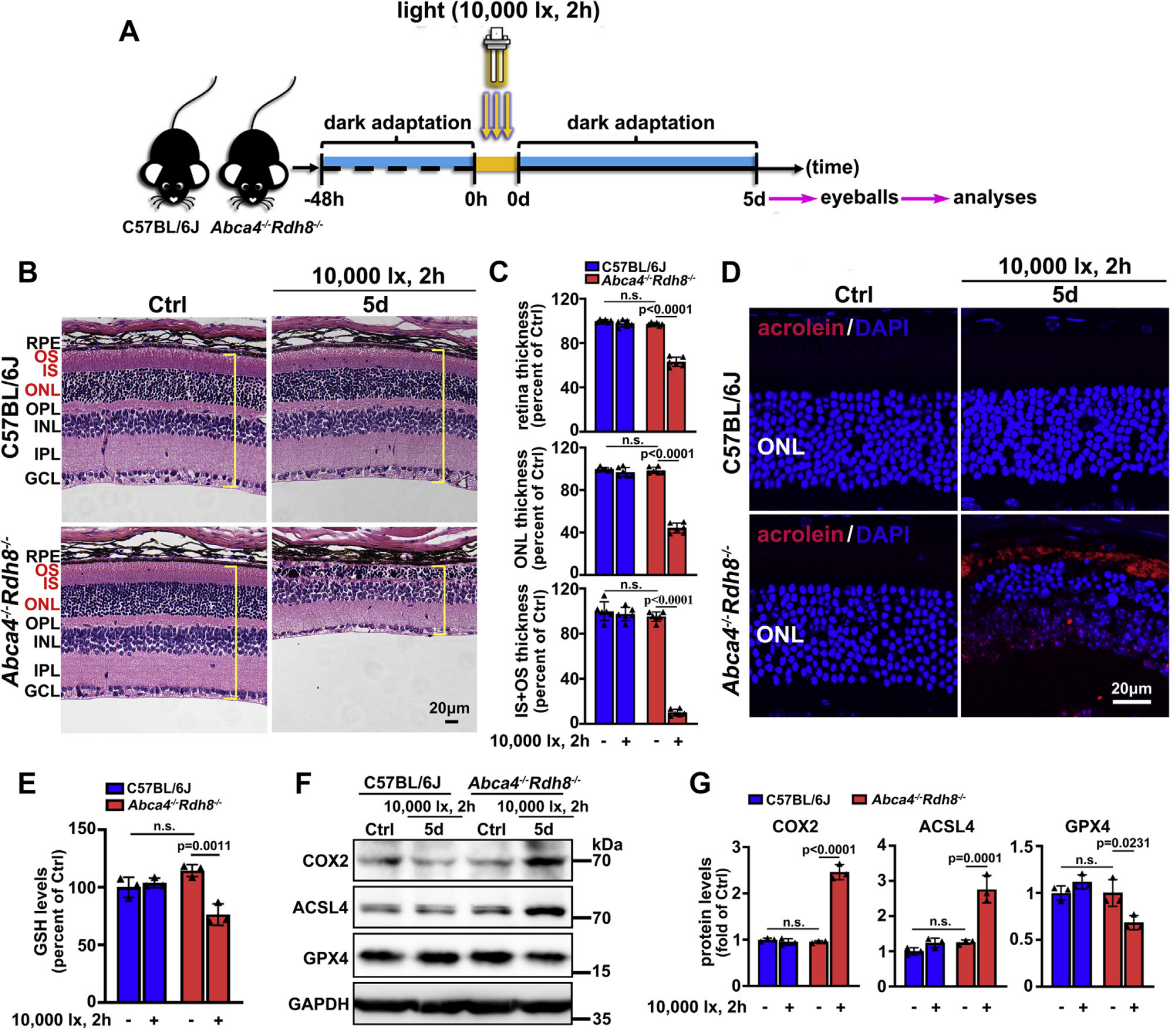
**Ferroptosis involves light-induced photoreceptor degeneration in *Abca4***^***−/−***^***Rdh8***^***−/−***^**mice.***A*, schematic illustration of the experimental procedure. Four-week-old C57BL/6J and *Abca4*^*−/−*^*Rdh8*^*−/−*^ mice were dark adapted and irradiated as we previously described (19). Eyeballs were harvested at day 5 after light exposure. *B*, histological examination of mouse retina was performed by using H&E staining. Square brackets in *yellow* indicate whole mouse retina. GCL, ganglion cell layer; INL, inner nuclear layer; IPL, inner plexiform layer; IS, inner segment; ONL, outer nuclear layer; OPL, outer plexiform layer; OS, outer segment; RPE, retinal pigment epithelium. *Scale bars*, 20 μm. *C*, thickness of whole retina, ONL, or IS+OS was quantified by Leica Application Suite X microscope software, and presented as a percentage of that measured in control C57BL/6J mice, respectively. *D*, lipid peroxidation in mouse retina was evaluated by immunofluorescence staining of lipid peroxidation marker acrolein. Nuclei were stained with DAPI (*blue*). *Scale bars*, 20 μm. *E*, GSH levels in mouse retina were determined by the GSH assay kit and expressed as a percentage of that detected in control C57BL/6J mice. *F*, western blot analysis of COX2, ACSL4, and GPX4 in lysates of mouse neural retina. *G*, protein levels of COX2, ACSL4, and GPX4 were normalized to those of GAPDH and presented as fold changes relative to control C57BL/6J mice. n.s., not significant.

As illustrated in Figure 8*A*, C57BL/6J and *Abca4*^*−/−*^*Rdh8*^*−/−*^ mice aged 4 weeks were dark adapted for 48 h, irradiated for 2 h by 10,000-lx light emitting diode (LED) light, and then raised in the dark for 5 days, respectively. Consistent with our most recent report (19), hematoxylin and eosin (H&E) staining indicated that neural retina from *Abca4*^*−/−*^*Rdh8*^*−/−*^ mice exposed to light visibly suffered from histological injury (Fig. 8*B*), and thicknesses of whole neural retina, outer nuclear layer (ONL), and inner and outer segments (OS+IS) were all decreased observably (Fig. 8, *B*–*C*). Acrolein, a product of lipid peroxidation reaction, is identified as a marker of lipid peroxidation (53, 54). Immunofluorescence staining for acrolein showed that lipid peroxidation was significantly aggravated in neural retina from *Abca4*^*−/−*^*Rdh8*^*−/−*^ mice upon light exposure (Fig. 8*D*). Interestingly, there was a clear downregulation of GSH levels in neural retina of light-exposed *Abca4*^*−/−*^*Rdh8*^*−/−*^ mice (Fig. 8*E*). As detected by western blotting, significant increases in protein expression of ferroptosis-related COX2 and ACLS4 were observed in neural retina of light-exposed *Abca4*^*−/−*^*Rdh8*^*−/−*^ mice (Fig. 8, *F*–*G*). Glutathione peroxidase 4 (GPX4), a selenoenzyme responsible for diminishing phospholipid hydroperoxide by oxidation of GSH, has been recognized as a biomarker of ferroptosis (23). As expected, immunoblot analysis displayed a distinct decline in protein levels of GPX4 in neural retina from *Abca4*^*−/−*^*Rdh8*^*−/−*^ mice exposed to light (Fig. 8, *F*–*G*). In contrast to light-exposed *Abca4*^*−/−*^*Rdh8*^*−/−*^ mice, no obvious photoreceptor degeneration and reduction in thickness of whole neural retina, ONL, or OS+IS were present in control *Abca4*^*−/−*^*Rdh8*^*−/−*^ and light-exposed C57BL/6J mice in comparison with control C57BL/6J mice (Fig. 8, *B*–*C*). Likewise, protein expression of COX2, ACSL4, and GPX4 as well as levels of GSH remains almost intact in neural retina of control *Abca4*^*−/−*^*Rdh8*^*−/−*^ and light-exposed C57BL/6J mice compared to control C57BL/6J mice (Fig. 8, *E*–*G*). Together the results hint that light-induced photoreceptor injury in *Abca4*^*−/−*^*Rdh8*^*−/−*^ mice at least partially correlates with ferroptosis and atRAL overaccumulation.

### Inhibition of ferroptosis by Fer-1 relieves photoreceptor degeneration in light-exposed *Abca4*^*−/−*^*Rdh8*^*−/−*^ mice

**Figure 9:**
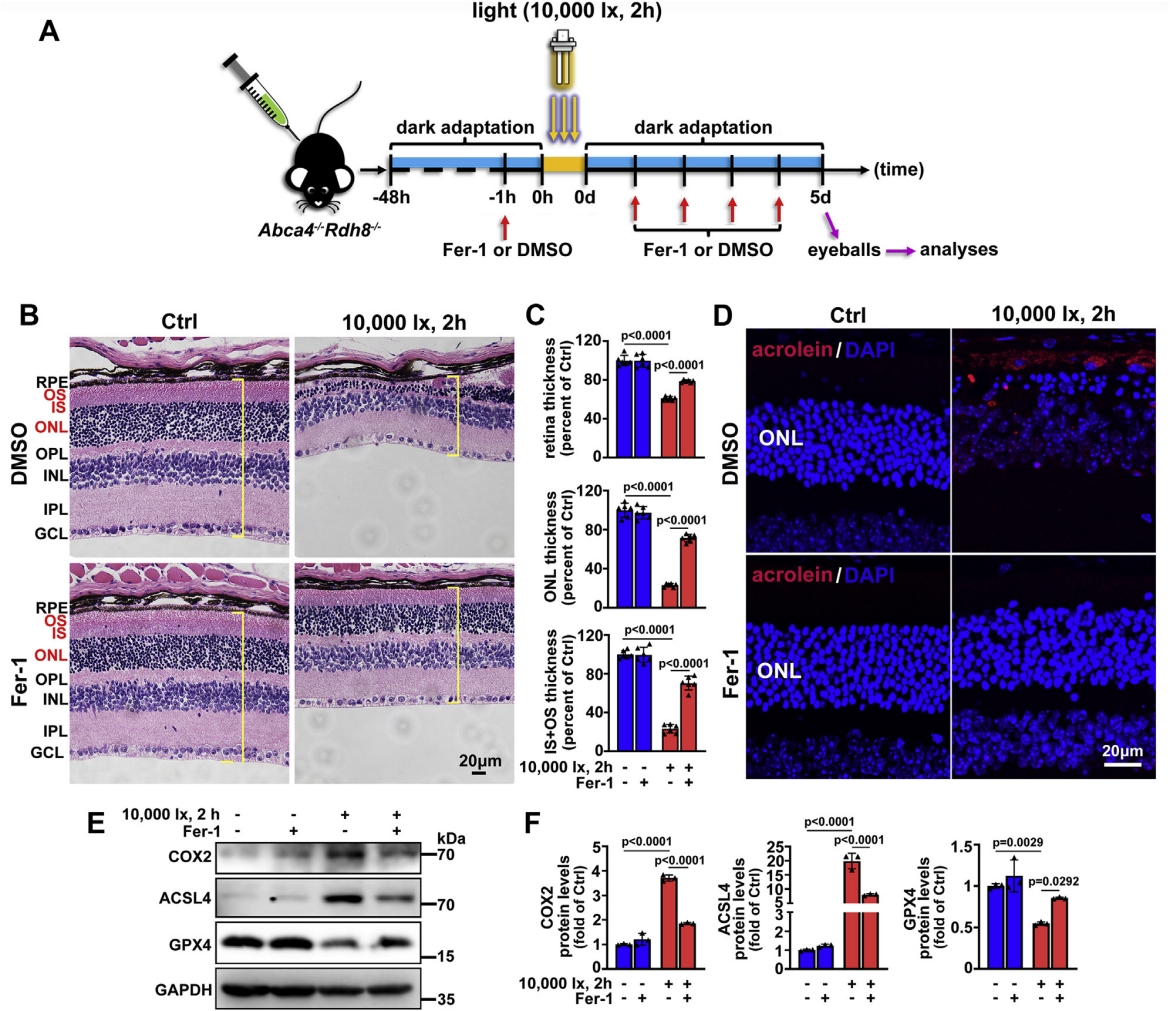
**Fer-1, a selective inhibitor of ferroptosis, alleviates light-induced photoreceptor degeneration and ferroptosis in *Abca4***^***−/−***^***Rdh8***^***−/−***^**mice.***A*, schematic diagram of the experimental protocol. *Abca4*^*−/−*^*Rdh8*^*−/−*^ mice aged 4 weeks were adapted in the dark for 48 h and then intraperitoneally injected with Fer-1 or vehicle (DMSO) (4 mg/kg body weight). After 1 h, pupils of mice were dilated with 1% tropicamide, and the mice were irradiated by 10,000-lx LED light for 2 h, followed by once-daily treatment with Fer-1 or vehicle (DMSO) (4 mg/kg body weight) in the dark for 4 days. Eyeballs, 5 days after light exposure, were harvested for subsequent analyses. Control *Abca4*^*−/−*^*Rdh8*^*−/−*^ mice were administered intraperitoneally Fer-1 (4 mg/kg body weight) or vehicle (DMSO) in the dark without exposure to light. *B*, H&E staining for analysis of the morphology of mouse retina, *Scale bars*, 20 μM. *C*, thickness of whole retina, ONL, or IS+OS was quantified by Leica Application Suite X microscope software and shown as a percentage of that measured in vehicle (DMSO)-treated control *Abca4*^*−/−*^*Rdh8*^*−/−*^ mice. *D*, lipid peroxidation in mouse retina was determined by immunofluorescence staining of lipid peroxidation marker acrolein. Nuclei were stained *blue* with DAPI. *Scale bars*, 20 μm. *E*, western blot analysis of COX2, ACSL4, and GPX4 in lysates of mouse neural retina. *F*, protein levels of COX2, ACSL4, and GPX4 were normalized to those of GAPDH and expressed as fold changes relative to vehicle (DMSO)-treated control *Abca4*^*−/−*^*Rdh8*^*−/−*^ mice.

Light-exposed *Abca4*^*−/−*^*Rdh8*^*−/−*^ mice were intraperitoneally injected with ferroptosis inhibitor Fer-1, as schematically shown in Figure 9*A*. Histological evaluation by H&E staining disclosed that intraperitoneal treatment with Fer-1 (4 mg/kg body weight) effectively ameliorated photoreceptor atrophy in *Abca4*^*−/−*^*Rdh8*^*−/−*^ mice exposed to light (Fig. 9*B*). Moreover, intraperitoneal administration of Fer-1 significantly prevented the reduction in the thickness of whole retina, ONL or IS+OS in light-exposed *Abca4*^*−/−*^*Rdh8*^*−/−*^ mice (Fig. 9, *B*–*C*). Immunofluorescence staining for lipid peroxidation marker acrolein demonstrated that intraperitoneal injection of Fer-1 clearly mitigated lipid peroxidation in neural retina of *Abca4*^*−/−*^*Rdh8*^*−/−*^ mice upon light exposure (Fig. 9*D*). To further confirm that ferroptotic cell death was indeed suppressed by Fer-1 in neural retina from light-exposed *Abca4*^*−/−*^*Rdh8*^*−/−*^ mice, protein expression of COX2, ACSL4, and GPX4 was examined by western blotting. As expected, Fer-1 administration remarkably decreased protein levels of COX2 and ACSL4, but clearly elevated GPX4 protein expression in neural retina of light-exposed *Abca4*^*−/−*^*Rdh8*^*−/−*^ mice (Fig. 9, *E*–*F*). These findings reveal that ferroptosis may serve as a potential target for rescuing photoreceptor degeneration against atRAL attack in *Abca4*^*−/−*^*Rdh8*^*−/−*^ mice.

## Discussion

As of yet, there are no approved effective therapies for dry AMD and STGD1. Dry AMD accounts for nearly 90% of all AMD cases (55), and in its advanced stage, geographic atrophy occurs and then leads to severe vision loss or even legal blindness (56, 57). STGD1, which is attributed to mutations in *Abca4* gene, is the most common hereditary macular dystrophy affecting children, with an estimated incidence of 1 in 10,000 (58). Children with STGD1 will go blind when they reach adulthood. Photoreceptor cell death is a fundamental cause of dry AMD and STGD1 (59, 60). Apoptosis of photoreceptor cells has been identified in a mouse model of dry AMD and STGD1 (19). Given that inhibiting apoptosis can only partially improve outcomes in mice subjected to photoreceptor degeneration (19), we wondered if another form of photoreceptor cell death is present in dry AMD and STGD1. In recent years, ferroptosis, an iron-dependent form of nonapoptotic cell death, has drawn increasing attention to researchers for its close association with cancer therapy (20, 61, 62), embryonic development (20), and several human diseases (30, 31, 32, 33, 34, 35, 36). Nevertheless, the relationship between ferroptotic cell death and photoreceptor atrophy in dry AMD and STGD1 is still ambiguous. *Abca4*^*−/−*^*Rdh8*^*−/−*^ mice, which are characteristic of dry AMD and STGD1, display a disruption in atRAL clearance and develop severe photoreceptor degeneration (9). Studies in the past have documented that exposure of *Abca4*^*−/−*^*Rdh8*^*−/−*^ mice to bright light leads to increased levels of atRAL in neural retina and causes serious damage to retinal photoreceptors (10, 19, 63). Herein, we confirmed the occurrence of ferroptosis in neural retina of *Abca4*^*−/−*^*Rdh8*^*−/−*^ mice *versus* age-matched C57BL/6J mice upon light exposure (Fig. 8, *D*–*G*). Obviously, these data suggest that atRAL accumulation activates photoreceptor ferroptosis *in vivo*.

It should be mentioned here that the concentration of atRAL utilized in this study is of physiological significance (19, 64, 65). The onset of ferroptosis involves upregulation of COX2 and ACSL4, both of which are widely accepted biomarkers of ferroptosis (23, 24, 26, 27, 32). Using cell-based assays, we demonstrated that treating 661W photoreceptor cells with 5-μM atRAL significantly enhanced mRNA levels of *Ptgs2* and *Acsl4* genes and protein levels of COX2 and ACSL4 (Fig. 1, *C*–*G*). Importantly, expression levels of COX2 and ACSL4 proteins were clearly elevated in neural retina from *Abca4*^*−/−*^*Rdh8*^*−/−*^ mice upon light exposure (Fig. 8, *F*–*G*). These is already evidence that ACSL4 activation stimulates lipid peroxidation and thereby leads to ferroptosis (26, 27, 32). By contrast, COX2 inhibitors do not dampen ferroptosis (23, 66), which suggests that increased expression of *Ptgs2* gene and its encoded protein COX2 may only represent ferroptosis onset.

Previous studies indicate that iron overload gives rise to ROS by the Fenton reaction and elicits ferroptosis through ROS-mediated lipid peroxidation (20). We have lately demonstrated that abundant ROS (including mitochondrial ROS) are generated in atRAL-loaded 661W photoreceptor cells (19). The results shown in Figures 2 and 3 confirmed that atRAL interrupted iron homeostasis to increase Fe^2+^ and dramatically induced lipid peroxidation in 661W photoreceptor cells. Reducing Fe^2+^ by iron-chelator DFO in atRAL-treated 661W photoreceptor cells clearly inhibited ROS production, significantly downregulated expression levels of *Ptgs2* and *Acsl4* genes and COX2 and ACSL4 proteins, and effectively enhanced cell survival (Fig. 6), thus corroborating that ferroptosis caused by atRAL in 661W photoreceptor cells is iron-dependent.

System Xc^−^ serves to produce GSH that maintains intracellular redox homeostasis through neutralizing ROS (67). Disruption of system Xc^−^ reduces intracellular levels of GSH, which in turn increases ROS production (50, 51). Some ferroptosis inducers such as erastin and sulfasalazine have been shown to cause intracellular GSH depletion and redox instability through blocking system Xc^−^ activity, accompanied by a compensatory increase in transcriptional levels of its core component, SLC7A11 (20, 52, 68). In neural retina of light-exposed *Abca4*^*−/−*^*Rdh8*^*−/−*^ mice, we observed that GSH levels were significantly decreased (Fig. 8*E*). Likewise, atRAL remarkably provoked GSH depletion in 661W photoreceptor cells, along with significant increases in the expression of *Slc7a11* gene and SLC7A11 protein (Fig. 4, *A*–*D*). In addition to diminishing ROS production, supplement of exogenous GSH clearly restrained atRAL-induced increases in mRNA levels of *Slc7a11*, *Ptgs2*, and *Acsl4* genes, and protein expression of SLC7A11, COX2, and ACSL4 in 661W photoreceptor cells, and dramatically improved cell viability (Fig. 4, *E*–*I*). Clearly, these results show that atRAL triggers photoreceptor ferroptosis *via* repressing system Xc^−^ activity. Glutathione peroxidase 4 (GPX4) is a selenocysteine-containing GSH peroxidase that prevents lethal lipid peroxidation in mammals (69, 70). Proper functioning of GPX4 is specifically required for the inhibition of ferroptosis by GSH (23). *In vitro* and mouse studies have indicated that suppressing GPX4 may favor cancer therapy by inducing ferroptosis (23). Our results manifested a significant downregulation of protein levels of GPX4 in neural retina of light-exposed *Abca4*^*−/−*^*Rdh8*^*−/−*^ mice (Fig. 8, *F*–*G*), suggestive of a critical role of the system Xc^−^/GSH/GPX4 axis in photoreceptor ferroptosis caused by atRAL.

There has been a recent report that mitochondria is an important organelle involving cysteine deprivation-induced ferroptosis *via* facilitating lipid peroxidation in cancer cells (71). As expected, mitochondria in atRAL-loaded 661W photoreceptor cells exhibited ferroptotic characteristic changes, including shrinkage, reduced or vanished crista, and aggregation around nuclei (Fig. 5). In recent time, we disclose the ability of atRAL to induce the production of mitochondrial ROS in 661W photoreceptor cells (19), implying that ROS generated inside mitochondria, at least in part, takes part in atRAL-induced lipid peroxidation and ferroptosis.

It has been shown that ferroptosis inhibitor Fer-1 protects cancer cells from erastin-induced ferroptosis through inhibition of lipid peroxidation (20, 21). Here, treatment of atRAL-loaded 661W photoreceptor cells with Fer-1 remarkably increased cell viability *via* blocking ROS production, lipid peroxidation, and COX2 and ACSL4 activation (Fig. 7). Moreover, the results of animal experiments revealed that intraperitoneal injection of Fer-1 clearly alleviated photoreceptor atrophy *via* suppressing lipid peroxidation and COX2 and ACSL4 activation and preventing the decline of GPX4 protein expression in *Abca4*^*−/−*^*Rdh8*^*−/−*^ mice subjected to light exposure (Fig. 9). Hence, pharmacologically inhibiting ferroptosis may help treat atRAL-associated retinopathies, such as dry AMD and STGD1.

To further ascertain whether Fer-1 also exerts an influence on photoreceptor apoptosis caused by atRAL, levels of cleaved caspase-3 and γH2AX proteins were examined by western blotting. Interestingly, treatment with 20-μM Fer-1 significantly attenuated protein levels of cleaved caspase-3 and γH2AX in atRAL-loaded 661W photoreceptor cells (Fig. S1). In addition, we found that Fer-1 administration by intraperitoneal injection decreased protein levels of cleaved caspase-3 and γH2AX in neural retina of *Abca4*^*−/−*^*Rdh8*^*−/−*^ mice upon light exposure (Fig. S2). These evidences, together with our previous findings (19), suggest that in addition to restricting ferroptosis, Fer-1, at least in part, inhibits apoptosis of retinal photoreceptors through defying the assault of atRAL. In contrast to a previous study that JNK affects erastin-induced ferroptosis in cancer cells (72), our recent findings indicate that JNK activation promotes atRAL-induced photoreceptor apoptosis (19), suggesting that JNK signaling may regulate both apoptosis and ferroptosis.

**Figure 10:**
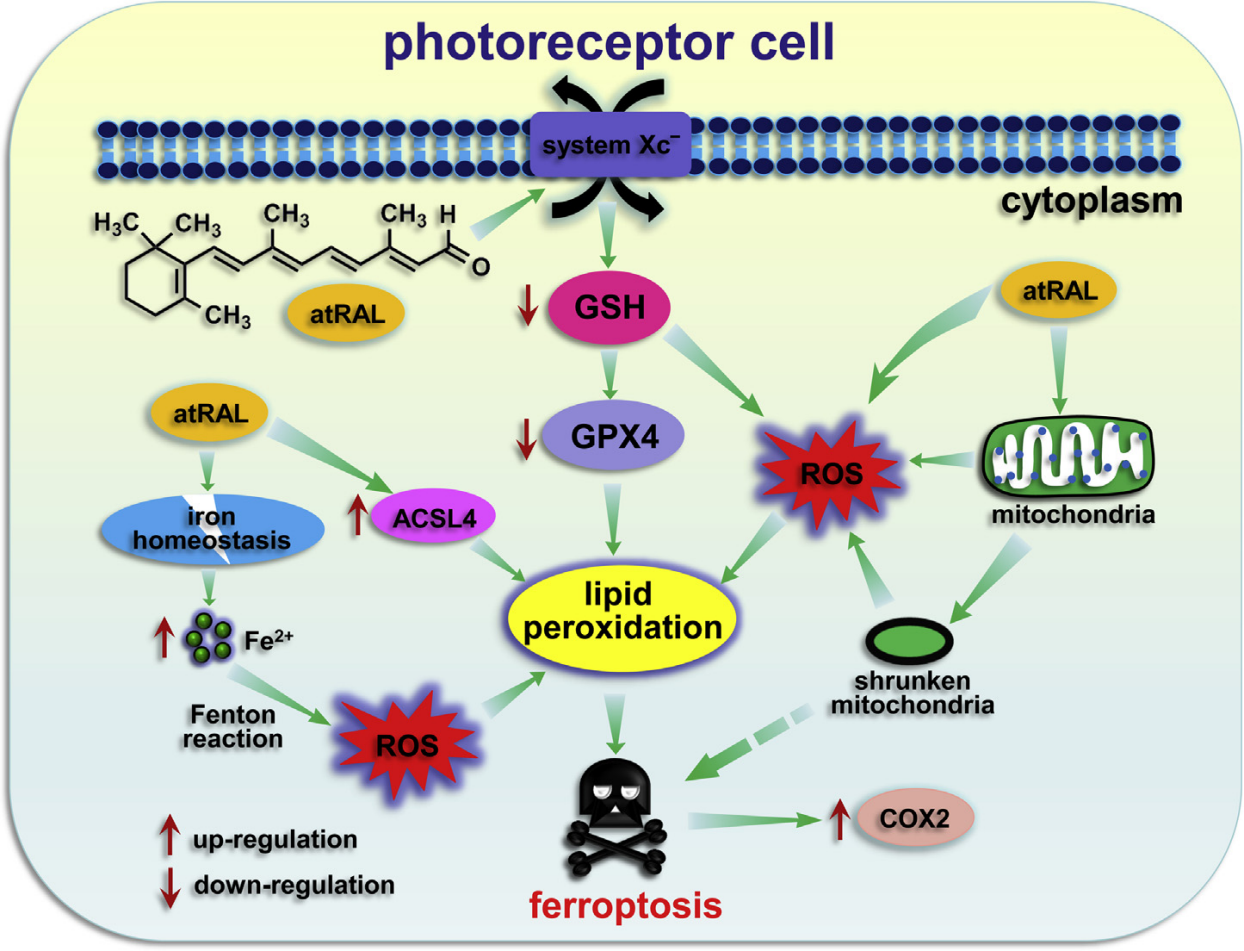
**Proposed mechanisms of photoreceptor ferroptosis induced by atRAL.** In photoreceptor cells, atRAL interrupts system Xc^−^ to elicit a significant decrease of GSH levels. GSH depletion inactivates GPX4 and produces ROS, which promotes lipid peroxidation and thereby induces ferroptosis. Mitochondrial ROS generated by atRAL (19) may also participate in lipid peroxidation. Damage to mitochondria inflicted by atRAL may directly evoke ferroptotic cell death. Besides increasing protein levels of ACSL4, atRAL elevates intracellular Fe^2+^ levels through perturbation of iron homeostasis and then provokes ROS production *via* Fenton reaction. Both ROS induced by overload of Fe^2+^ and activation of ACSL4 incite lipid peroxidation that triggers ferroptosis. In addition, atRAL stimulates a remarkable increase in protein levels of COX2 encoded by *Ptgs2* gene. Up-regulation of COX2 is a hallmarker of ferroptosis.

Based on foregoing results, we proposed a plausible mechanism for how atRAL mediates ferroptosis of photoreceptor cells. As depicted in Figure 10, bleaching of rhodopsin by light releases atRAL inside photoreceptor disk membranes, and it enters into the cytoplasm of photoreceptor cells and then accumulates overly largely. Disruption of system Xc^−^ by atRAL results in GSH depletion and subsequent GPX4 inactivation, thereby inducing ferroptosis by increasing lipid peroxidation. The depletion of GSH evokes ROS production, which promotes lipid peroxidation contributing to ferroptosis. Note that mitochondrial ROS generation induced by atRAL (19) may also serve as a source of ROS leading to lipid peroxidation. It is also likely for atRAL to elicit ferroptotic cell death by directly destructing mitochondria. In addition to activating ACSL4, atRAL enhances intracellular levels of Fe^2+^ *via* perturbing iron homeostasis and then provokes ROS production through Fenton reaction. Both ACSL4 activation and Fe^2+^-induced ROS stimulate lipid peroxidation able to incite ferroptosis. Furthermore, atRAL initiates the activation of COX2 encoded by *Ptgs2* gene, a hallmarker of ferroptosis. The results of this study lead us to propose that ferroptosis plays an important role in photoreceptor damage by the buildup of atRAL. Repression of different types of cell death by one or combined inhibitors may be benefit to the therapy of photoreceptor degeneration in patients with dry AMD and STGD1.

## Experimental procedures

### Materials

atRAL, Fer-1 (catalog no. SML0583), DFO (catalog no. D9533), 4',6-diamidino-2-phenylindole (DAPI) and Hoechst 33342 were purchased from Sigma-Aldrich (Saint Louis, MO, USA). 2',7'-dichlorodihydrofluorescein diacetate (H2DCFDA) and BODIPY 581/591 C11 (C11-BODIPY) were obtained from ThermoFisher Scientific (Eugene, OR, USA). Mitotracker Red CMXRos and TRIeasy total RNA extraction reagent were obtained from Yeasen (Shanghai, China). Antibodies against COX2 (catalog no. 12282S), SLC7A11 (catalog no. 98051S), cleaved caspase-3 (catalog no. 9664S and 9661S), and GAPDH (catalog no. 5174S) were provided by Cell Signaling Technology (Danvers, MA, USA). Anti-GPX4 (catalog no. ab125066), anti-ACSL4 (catalog no. ab155282), and anti-acrolein (catalog no. ab48501) were purchased from Abcam (South Cambs, England, UK). Anti-γH2AX (catalog no. 05-636) was purchased from Millipore (Billerica, MA, USA). Anti-γH2AX (catalog no. NB100-384) was provided by Novus Biologicals (Littleton, CO, USA).

### Animals

*Abca4*^*−/−*^*Rdh8*^*−/−*^ mice on a C57BL/6J genetic background and C57BL/6J mice were used in this study (19). Animal studies were approved by the Institutional Animal Care and Use Committee of Xiamen University School of Medicine. The mice at 4 weeks of age were dark adapted for 48 h and then illuminated for 2 h by 10,000-lx LED light (19). Eyeballs were harvested at day 5 after light exposure. Control mice were kept normally in the dark for 7 days without light exposure. Alternatively, *Abca4*^*−/−*^*Rdh8*^*−/−*^ mice aged 4 weeks were dark adapted for 48 h and then injected intraperitoneally with Fer-1 or vehicle (DMSO) at a dose of 4 mg/kg body weight. One hour later, the resulting mice were exposed to 10,000-lx LED light for 2 h, followed by once-daily administration of Fer-1 or vehicle (DMSO). Eyeballs were collected at day 5 after light illumination. Control *Abca4*^*−/−*^*Rdh8*^*−/−*^ mice were intraperitoneally administered with Fer-1 or vehicle (DMSO) alone without light illumination.

### H&E staining

Mouse eyeballs were fixed in paraformaldehyde at 4 °C for 2 days and then embedded in paraffin. Five-micrometer sections were cut from paraffin-embedded tissues and routinely stained with H&E. Images were examined using a DM2500 microscope (Leica; Wetzlar, Germany).

### Cell culture, viability, and morphology

Murine photoreceptor cell line 661W was obtained from Shanghai Zishi Biotechnology (Shanghai, China) and routinely cultured as previously described (19). 661W photoreceptor cells were treated for 3 and 6 h with atRAL at concentrations of 2.5, 5, 10, and 20 μM. Alternatively, cells were preincubated with GSH (2 and 4 mM) for 1 h, iron chelating agent DFO (50, 100, and 200 μM) for 2 h, or ferroptosis inhibitor Fer-1 (10 and 20 μM) for 2 h, followed by treatment with or without 5-μM atRAL for 6 h. Cells exposed to atRAL or vehicle (DMSO) alone served as controls. Cell viability was detected as previously reported (19). Cell morphology was photographed under a research-grade inverted microscope (Leica Microsystems; Wetzlar, Germany).

### Lactate dehydrogenase (LDH) release assay

LDH release assay was performed by using a LDH cytotoxicity assay kit (Yeasen; Shanghai, China) (73).

### Iron assay

661W photoreceptor cells cultured in 35-mm glass bottom dish with 15-mm micro-well chambered cover glass were treated with 5-μM atRAL for 3 and 6 h, or with vehicle (DMSO) alone for 6 h. Alternatively, cells were pretreated with 200-μM DFO for 2 h and incubated with or without 5-μM atRAL for 6 h. Cells treated for 6 h with atRAL or vehicle (DMSO) alone served as controls. On the one hand, the cells were exposed to 5-μM FeRhoNox-1 (Goryo Chemical; Sapporo, Japan) and 10-μM Hoechst 33342 for 1 h at 37 °C. After being washed three times with phosphate-buffered saline (PBS), 1-ml FBS-free DMEM was added into each well. To visualize intracellular Fe^2+^, the cells were then imaged with a Zeiss LSM 880+airy scan confocal microscope (Jena, Germany). The fluorescence intensity reflecting Fe^2+^ levels was quantified by ImageJ software. On the other hand, total iron levels in each sample were measured by ICP-MS. In brief, cell pellets were resuspended in 65% nitric acid and then incubated overnight at room temperature. Quantitative analyses of iron were made using a PerkinElmer NexION 2000 ICP-MS instrument.

### GSH levels assay

GSH levels were measured by a GSH assay kit (Beyotime; Shanghai, China) according to the manufacturer’s instructions. 661W photoreceptor cells seeded in 6-well plates were treated with 5-μM atRAL for 3 and 6 h or with vehicle (DMSO) for 6 h. After being washed twice with ice-cold PBS, the cells were harvested in chilled PBS by scraping. Following centrifugation at 1000 rpm for 5 min at 4 °C, the supernatant was removed, and the pellet was resuspended in appropriate amount of protein removal reagent M. Then, the cells underwent 2 freeze-and-thaw cycles using liquid nitrogen and a 37 °C water bath, respectively. The samples were placed in an ice bath for 5 min and then centrifuged at 10,000*g* for 10 min at 4 °C. The supernatant was collected into a 96-well plate, followed by inclusion of 150-μl total glutathione assay reagent in each well. After a 5-min incubation at room temperature, 50-μl nicotinamide adenine dinucleotide phosphate (NADPH) (0.5 mg/ml) was added into each well. The resulting solutions were spectrophotometrically measured at 412 nm.

### Transmission electron microscopy (TEM)

661W photoreceptor cells seeded in 6-well plates were incubated with 5-μM atRAL for 3 and 6 h or with vehicle (DMSO) for 6 h. The cells were digested by 0.25% trypsin-EDTA solution (Gibco; Shanghai, China), collected in a sterile 1.5-ml tube, and fixed with 3% glutaraldehyde solution overnight at 4 °C. Samples were embedded in Spurr’s resin, followed by ultrathin sectioning. Images were taken by a Hitachi HT-7800 TEM (Tokyo, Japan). Quantification of intact or shrunken/destructed mitochondria was carried out for at least three different fields at each time point, and the percentage was calculated as the number of shrunken/destructed mitochondria divided by total number of mitochondria in each field.

### Staining of mitochondria

661W photoreceptor cells seeded in 6-well glass bottom plates were treated with 5-μM atRAL for 3 and 6 h, or with vehicle (DMSO) for 6 h, and then incubated with 500-nM Mitotracker Red CMXRos for 15 min at 37 °C. After being washed with PBS three times, 500-μl FBS-free DMEM was added into each well, and the cells were photographed by a Zeiss LSM 880+airy scan confocal microscope (Carl Zeiss; Jena, Germany).

### Measurement of ROS production

661W photoreceptor cells seeded in 6-well plates were incubated with 5-μM atRAL for 6 h or with vehicle (DMSO) alone for 6 h. Alternatively, cells were preincubated with 4-mM GSH for 1 h, 200-μM DFO for 2 h, or 20-μM Fer-1 for 2 h, followed by treatment with or without 5-μM atRAL for 6 h, respectively. Then, the cells were treated with 10-μM H2DCFDA and 10-μM Hoechst 33342 for 20 min at 37 °C. After being washed three times in PBS, 500-μl FBS-free DMEM was added into each well, and the cells were observed under a DMi8 Leica fluorescence microscope (Leica Microsystems; Wetzlar, Germany).

### Lipid peroxidation assay

Lipid peroxidation was assessed using a Click-iT lipid peroxidation imaging kit (ThermoFisher Scientific; Rockford, IL, USA). 661W photoreceptor cells seeded on 15-mm glass slides in a 24-well plate were pretreated with 50-μM Click-it LAA for 30 min at 37 °C and then incubated with 5-μM atRAL for 3 and 6 h, 100-μM cumene hydroperoxide (CH) for 6 h, or vehicle (DMSO) alone for 6 h at 37 °C. Alternatively, cells were pretreated with 20-μM Fer-1 for 2 h at 37 °C and incubated with 50-μM Click-it LAA for 30 min, followed by treatment with or without 5-μM atRAL for 6 h, respectively. Cells treated with atRAL or vehicle (DMSO) alone served as controls. Subsequently, the cells were fixed with 4% paraformaldehyde for 15 min at room temperature, washed three times with PBS, permeabilized by a 0.5% Triton X-100, and then blocked with 1% BSA. After 50-μl Click-iT reaction cocktail containing 5-μM Alexa Fluor 488 was added into each slide, the cells were incubated for 30 min at 37 °C in the dark, stained with DAPI, and then examined by using an Olympus FV1000 confocal microscope (Tochigi, Japan). To further quantify lipid peroxidation, levels of lipid peroxides were evaluated by flow cytometry using C11-BODIPY dye (74). Samples were subjected to a Beckman Coulter Gallios flow cytometer (Brea, CA, USA), and data were analyzed using FlowJo 10 software (TreeStar; Ashland, OR, USA).

### Immunofluorescence staining

Immunofluorescence staining was performed as previously reported (54). Briefly, tissue sections from mouse eyeballs were incubated with the anti-acrolein antibody (1:200 dilution) at 4 °C overnight, followed by incubation for 2 h with Alexa Fluor 594-conjugated donkey anti-mouse (1:200 dilution) secondary antibody at room temperature. Nuclei were stained by DAPI. The photographs were acquired by the Olympus FV1000 confocal microscope.

### qRT-PCR

TRIeasy Total RNA Extraction Reagent was used to isolate total RNA from cultured cells, and 2 μg of total RNA served as a template for cDNA synthesis. Reverse transcription of RNA to cDNA was performed by a ReverTra Ace qPCR RT Master Mix kit (Toyobo; Osaka, Japan), and qRT-PCR was determined using a LightCycler 96 instrument (Roche; Basel, Switzerland). The value of each target gene was normalized relative to that of *Gapdh*. The sequences of qRT-PCR primers were obtained from NCBI’s primer designer and shown in Table 1. All primers were synthesized by Sangon Biotech (Shanghai, China).

**Table 1:** Primer sequences for qRT-PCR

Gene	Species	Forward primer (5′→3′)	Reverse primer (5′→3′)
*Ptgs2*	mouse	AATGTATGAGCACAGGATTTGACC	TGTCAGCACATATTTCATGATTAAACTTCG (75)
*Acsl4*	mouse	GCTATGACGCCCCTCTTTGT	GAATCGGTGTGTCTGAGGGG
*Slc7a11*	mouse	GGTCAGAAAGCCAGTTGTGG	AGTATGCCCTTGGGGGAGAT
*Tf*	mouse	CCAGAGGGTACCACACCTGA	TCCAGGAGTCGTGAGGTTGA
*Tfrc*	mouse	CTCAGTTTCCGCCATCTCAGT	GCAGCTCTTGAGATTGTTTGCA
*Steap3*	mouse	GATGTAAGCAACCCCACGGA	AGATGAGCACCTGCCTGTTC
*Dmt1*	mouse	AGGATGTGGAGCACCTAACG	AACTCACTCATCACTGGCCG
*Ireb2*	mouse	GTGACACTGTCTCTGTTCGT	TGTGTAACCATCCCACTGCC (75)
*Fth1*	mouse	CGGGCCTCCTACACCTACCT	CCCTCCAGAGCCACGTCAT
*Ftl1*	mouse	GGAGCGTCTCCTCGAGTTTC	CAGGGCATGCAGATCCAAGA
*Ncoa4*	mouse	CACGCGAGCTCCTCAAGTAT	GTCCTGTGGGTTGGTACTGG
*Cp*	mouse	CTATGTGGCAGCCGTAGAGG	GGCTCGTCTCTTCACCTGTT
*Heph*	mouse	AAGGGACATTGGAGGTGACAA	AGCCAGAGCAATGAGCAACA
*Fpn*	mouse	GTCTCTGTCAGCCTGCTGTT	CTTGCAGCAACTGTGTCACC
*Gapdh*	mouse	AGGTCGGTGTGAACGGATTTG	TGTAGACCATGTAGTTGAGGTCA (75)

### Western blotting

Immunoblot analysis of extracts from cells and neural retina tissues was performed as previously described (19).

### Statistical analysis

GraphPad Prism software (Version 8.0; La Jolla, CA, USA) was used for analysis of the results. All data were presented as the mean ± standard deviation (SD) of at least three independent experiments. Statistical analyses for the data of cell-based experiments were carried out using one-way of variance (ANOVA) followed by Tukey’s multiple comparison test. The results of animal studies were statistically analyzed by two-way ANOVA followed by Sidak’s multiple comparison test. In all cases, *p*-values below 0.05 were considered statistically significant.

## Data availability

The data supporting the findings of this study are available within the article and the supporting information.

## Conflicts of interest

The authors declare that they have no competing conflicts of interest with the contents of this article.
