# Supplementary material for: Ferroptosis drives photoreceptor degeneration in mice with defects in all-trans-retinal clearance
Source: J Biol Chem. 2020 Dec 20;296:100187. doi: 10.1074/jbc.RA120.015779 (PMC7948481; doi:10.1074/jbc.RA120.015779)
Supplement: Figures S1 and S2 [file mmc1.pdf]

Supplementary Information

Ferroptosis drives photoreceptor degeneration in mice with defects in all-*trans*-retinal clearance

Chao Chen[1], Jingmeng Chen[2], Yan Wang[3], Zuguo Liu[1], and Yalin Wu[1,4,5]*

[1]Department of Ophthalmology, Xiang'an Hospital of Xiamen University, Fujian Provincial Key Laboratory of Ophthalmology and Visual Science, Eye Institute of Xiamen University, School of Medicine, Xiamen University, Xiamen City, FJ 361102, China
[2]School of Medicine, Xiamen University, Xiamen City, FJ 361102, China
[3]Department of Ophthalmology, Shenzhen Hospital, Southern Medical University, Shenzhen City, GD 518100, China
[4]Xiamen Eye Center of Xiamen University, Xiamen City, FJ 361001, China
[5]Shenzhen Research Institute of Xiamen University, Shenzhen City, GD 518063, China

*To whom correspondence should be addressed: Yalin Wu, Xiang'an Hospital of Xiamen University, Eye Institute of Xiamen University, School of Medicine, Xiamen University, Xiang'an South Road, Xiang'an District, Xiamen City, FJ, China; or Xiamen Eye Center of Xiamen University, Wutong West Road, Huli District, Xiamen City, FJ, China; E-mail: yalinw@xmu.edu.cn

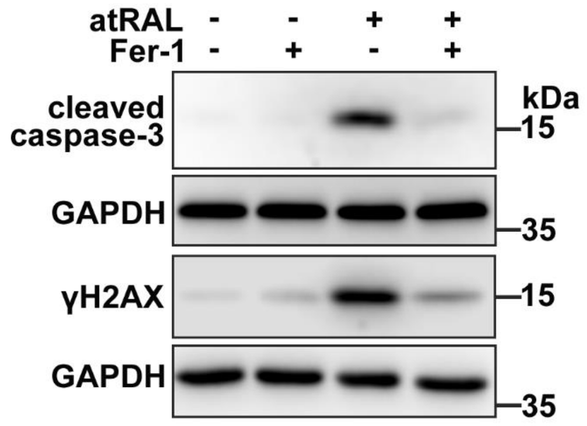

Supplementary Figure S1. Treatment with Fer-1 attenuates protein levels of cleaved caspase-3 and γH2AX in atRAL-loaded 661W photoreceptor cells. Cells were pretreated with 20-μM Fer-1 for 2 h and then incubated with 5-μM atRAL for 6 h. Control cells were exposed to atRAL, Fer-1 or vehicle (DMSO) alone. Molecular mass markers (kDa) were indicated to the *right* of immunoblots. GAPDH served as loading controls.

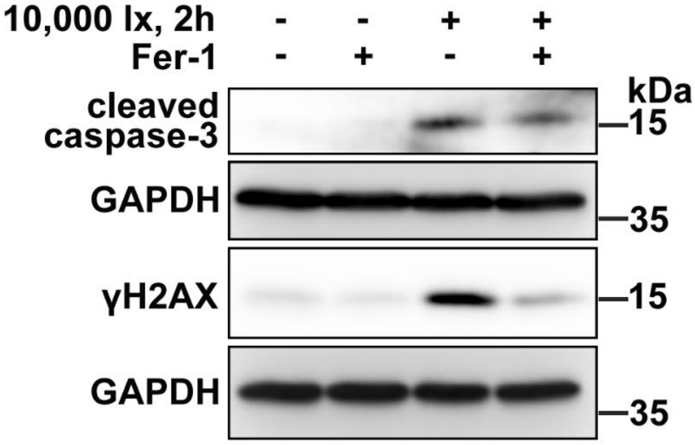

Supplementary Figure S2. Intraperitoneal administration of Fer-1 decreases protein levels of cleaved caspase-3 and γH2AX in neural retina of *Abca4*$^{-/-}$*Rdh8*$^{-/-}$ mice upon light exposure. Either Fer-1 or vehicle (DMSO) (4 mg/kg body weight) was intraperitoneally administered after *Abca4*$^{-/-}$*Rdh8*$^{-/-}$ mice at 4 weeks of age were adapted in the dark for 48 h. After an hour, pupils of mice were dilated with 1% tropicamide, and the mice were illuminated by 10,000-lx LED light for 2 h, followed by once-daily treatment with Fer-1 or vehicle (DMSO) in the dark for 4 days. Eyeballs, 5 days after light exposure, were harvested, and neural retinas were isolated for analyzing protein levels of cleaved caspase-3 and γH2AX by Western blotting. Control *Abca4*$^{-/-}$*Rdh8*$^{-/-}$ mice were injected intraperitoneally with Fer-1 or vehicle (DMSO) in the dark without exposure to light. Molecular mass markers (kDa) were indicated to the *right* of immunoblots. GAPDH served as internal controls.
